# Digital Detox at Sea: Reducing Internet and Smartphone Overuse while Improving Well-being in Young Adults

**DOI:** 10.1192/j.eurpsy.2026.11819

**Published:** 2026-07-31

**Authors:** M. W. Romaniuk

**Affiliations:** The Maria Grzegorzewska University, Warsaw, Poland

## Abstract

**Introduction:**

Problematic use of internet and smartphones has become a significant risk factor for mental health, particularly among young adults. Excessive screen time and compulsive digital behaviors are linked to decreased psychological well-being, impaired emotion regulation, and reduced social connectedness. Nature- and adventure-based interventions, combined with enforced digital abstinence, may represent an innovative strategy for mental health promotion. Sailing voyages on traditional tall ships provide a unique environment of teamwork, physical activity, and complete disconnection from digital media.

**Objectives:**

The study aimed to examine whether a month-long tall ship voyage, characterized by complete digital detox, leads to a measurable reduction in compulsive internet and smartphone use, and to evaluate concomitant changes in psychological well-being.

**Methods:**

A repeated-measures design with five assessment points (Day 1, Day 8, Day 16, Day 26, and one-month follow-up) was applied to a convenience sample of 20 young adults (aged 18–26 years) participating in a 27-day sailing expedition from Gdańsk (Poland) to Longyearbyen (Svalbard). Standardized self-report instruments were administered: Compulsive Internet Use Scale (CIUS-14), Smartphone Addiction Scale Short Version (SAS-SV-10), WHO-5 Well-being Index, and PERMA-23 Profiler. Statistical analyses will include repeated-measures ANOVA and linear mixed-effects models to assess time effects, with effect size estimation and post-hoc contrasts.

**Results:**

It is hypothesized that participants will demonstrate significant decreases in CIUS and SAS-SV scores across the voyage, accompanied by progressive improvements in WHO-5 and PERMA scores. The strongest changes are expected between baseline and the end of the voyage, with sustained effects at one-month follow-up.

**Conclusions:**

If confirmed, these findings would provide empirical support for tall ship sailing as an effective preventive and health-promoting blue intervention, integrating digital detox, exposure to restorative natural environments, and collective experiential learning.

**Disclosure of Interest:**

None Declared

